# Contamination of imported kernels by unapproved genome-edited varieties poses a major challenge for monitoring and traceability during transport and handling on a global scale: inferences from a study on feral oilseed rape in Austria

**DOI:** 10.3389/fgeed.2023.1176290

**Published:** 2023-04-20

**Authors:** Kathrin Pascher, Christa Hainz-Renetzeder, Michaela Jagersberger, Katharina Kneissl, Günter Gollmann, Gerald M. Schneeweiss

**Affiliations:** ^1^ Department of Botany and Biodiversity Research, University of Vienna, Vienna, Austria; ^2^ Institute of Zoology, Department of Integrative Biology and Biodiversity Research, University of Natural Resources and Life Sciences, Vienna, Austria; ^3^ Institute of Landscape Development, Recreation and Conservation Planning (ILEN), Department of Landscape, Spatial and Infrastructure Sciences, University of Natural Resources and Life Sciences, Vienna, Austria; ^4^ Department of Evolutionary Biology, Unit for Theoretical Biology, University of Vienna, Vienna, Austria

**Keywords:** genome-edited plants, risk assessment, seed spillage, *Brassica napus*, genetic diversity

## Abstract

Novel techniques such as CRISPR/Cas are increasingly being applied for the development of modern crops. However, the regulatory framework for production, labelling and handling of genome-edited organisms varies worldwide. Currently, the European Commission is raising the question whether genome-edited organisms should still be regulated as genetically modified organisms in the future or whether a deregulation should be implemented. In our paper, based on the outcome of a 2-year case study on oilseed rape in Austria, we show that seed spillage during import and subsequent transport and handling activities is a key factor for the unintended dispersal of seeds into the environment, the subsequent emergence of feral oilseed rape populations and their establishment and long-term persistence in natural habitats. These facts must likewise be considered in case of genome-edited oilseed rape contaminants that might be accidentally introduced with conventional kernels. We provide evidence that in Austria a high diversity of oilseed rape genotypes, including some with alleles not known from cultivated oilseed rape in Austria, exists at sites with high seed spillage and low weed management, rendering these sites of primary concern with respect to possible escape of genome-edited oilseed rape varieties into the environment. Since appropriate detection methods for single genome-edited oilseed rape events have only recently started to be successfully developed and the adverse effects of these artificial punctate DNA exchanges remain largely unknown, tracing the transmission and spread of these genetic modifications places high requirements on their monitoring, identification, and traceability.

## 1 Introduction

New genomic plant breeding techniques or in short ‘new techniques’ (SAM, 2017) - in particular, genome-editing tools such as Clustered Regularly Interspaced Short Palindromic Repeats/Cas system (CRISPR/Cas) - promise to be simple, user-friendly, precise, efficient, versatile, cost-effective, and time saving (Stout et al., 2017). However, these new techniques face several technical limitations and challenges (Kawall et al., 2020). Although the genetic interventions target short regions of the genome, in some cases only one base pair, they may have large effects on the enhancement of genes in their activity or phenotypic expression. Application of this method has shown increased off-target effects (Yee, 2016; Eckerstorfer et al., 2019). Another point of criticism is that these new techniques have always been evaluated as techniques *per se* without considering genome-edited organisms in the context of cropping systems (Hilbeck, 2022) and potential adverse effects on the receiving environment. Accordingly, detailed ecological risk assessment and long-term experience are still missing (Gelinsky and Hilbeck, 2018; Eckerstorfer et al., 2019; 2021).

For cultivation and trade of genome-edited organisms differing legal regulations are in place worldwide. Europe currently follows a *process-oriented* assessment regarding the regulation of genome-edited organisms and products (Schuler et al., 2019), whereas, e.g., the US, Canada, and Australia follow *product-oriented* assessment (Medvedeva and Blume, 2018)*.* In the European Union, the Directive 2001/18/EC of the European Parliament and of the Council of 12 March 2001 on the deliberate release into the environment stipulates that the potential adverse effects on the protection of *human health* and *environment* must undergo a predetermined risk assessment before their approval and release (European Commission, 2001). On 25 March 2018, the European Court of Justice decided that in this context genome-edited organisms must be treated in the same way as classical genetically modified organisms (GMOs) (Court of Justice of the European Union. InfoCuria, 2018). Among other aspects, the demanding requirements of traceability, especially in connection with international trade activities (e.g., Ribarits et al., 2021a; Ribarits et al., 2021b), have triggered renewed discussions on whether or not a genome-edited organism should continue to be treated as a classical GMO (e.g., Winter 2019; van der Meer et al., 2020). Despite the existing establishment of strict approval procedures in Europe, a decision on the possible deregulation of genome-edited organisms is pending and expected to be announced from the European Commission in 2023. There is a possibility that the strict European legislation on genetic engineering could be weakened in near future.

At present, three genome-edited crop species—soybean from U.S. company Calyxt, “waxy corn” produced by a company Corteva based in the United States, and canola (synonym for oilseed rape: OSR) from United States company Cibus US LLC—are cultivated and placed on the market in the US, Canada, Argentina, Brazil, and Chile (https://grain.org/, accessed on 19 March 2023). However, detailed data on the cultivation area extent are still not available. One genome-edited OSR variety carries event 5715, which provides increased herbicide tolerance to imidazolinone and sulfonylurea herbicides compared to conventionally produced varieties (Health Canada, 2016). In addition, another genome-edited variety contains event 5720, which is a re-transformation or re-mutation of event 5715. There is a realistic possibility that Cibus genome-edited OSR—although not approved in the EU—could enter EU countries along transport routes via contaminated imported kernels. For the Cibus herbicide-tolerant genome-edited OSR variety, open source event-specific detection tests have existed since 2020 (Chhalliyil et al., 2020; Chhalliyil et al., 2022; https://www.detect-gmo.org/ accessed on 19 November 2022). With a PCR test, the artificial point mutation could be identified, and moreover, its share in a sample could be calculated. However, according to the German Federal Office of consumer Protection and Food Safety (BVL, 2021), a confirmed detection of the genome-edited Cibus OSR is still in question. Tools like this would mark a huge improvement in the transparency process in the non-GMO—organic and conventional—food production chain of EU countries and would further facilitate detection procedures in the context of kernel import.

The methodological detection of most other genome-edited products, however, still remains a major hurdle, especially considering that a genome-edited organism may have multiple targeted point mutations, rendering detection of multiple events particularly challenging. However, evidence has been compiled in a number of publications that could enable the detection of genome-edited products or even identify the techniques behind their development (Bertheau, 2019; 2022; Ribarits et al., 2021a; Ribarits et al., 2021b; Ribarits et al., 2022; Fraiture et al., 2022; Potthof et al., 2023). In addition, initiatives such as the Norwegian Foodprint program NORCE (https://foodprintproject.org/; accessed on 21 March 2023) aim to develop gene-editing detection tools for traceability and labelling of genetically modified (GM) products throughout the food chain.

For the OSR crop, additional plant-specific characteristics have to be considered for monitoring of kernels that may illegally contain genome-edited seeds imported into countries where these events are not authorized. For this reason, there are special requirements for the monitoring of OSR resulting from factors such as high seed spillage, persistent soil seed banks, and high proportion of gene flow to closely related wild species (Chevré et al., 2004; Adamczyk-Chauvat et al., 2017). In addition, contamination of conventionally or organically grown crops in the field (Andersen et al., 2010; Areal et al., 2015) is likely to occur, which could affect compliance with coexistence requirements (Bailleul et al., 2016). In this context, unauthorized genome-edited OSR seeds could be spread during handling and transport activities, resulting in proliferation of feral OSR populations containing the artificial modification. Their emergence and establishment in natural habitats would pose a major risk to the environment (e.g., Mizuguti et al., 2011; Schoenenberger and D´Andrea, 2012; Hecht et al., 2013). To shed more light on this issue, we use sample data from a 2-year study on OSR in Austria (Pascher et al., 2016; Pascher et al., 2017) to illustrate the potential for accidental contamination of imported conventional kernels with genome-edited seeds and their spillage, appearance and proliferation as plants in feral populations.

Oilseed rape (OSR; *Brassica napus*) is an ancient, currently worldwide cultivated crop that probably originated from a unique breeding event of *Brassica oleracea* (cabbage) and *Brassica rapa* (turnip) in Europe (Gómez-Campo and Prakash, 1999; Allender and King, 2010). Due to the absence of erucic acid and glucosinolates, today OSR is frequently cultivated in Europe for use as a source of edible oil, animal feed stock (press cake, grid), and renewable raw material for bio-fuel (Moser et al., 2013). The crop OSR is unknown as a wild plant but frequently becomes feral. Feral plants can reproduce and populations can persist for many years. As several closely related species of the family Brassicaceae are known to successfully hybridize with OSR (Pascher and Gollmann, 1999; Chevré et al., 2004), feral OSR populations could function as a bridge for transgenes or artificial mutations from GM OSR varieties including genome editing into semi-natural and natural plant communities.

Seed spillage during transport is a crucial factor for the establishment and persistence of feral OSR populations along transport routes, for instance in France (Pessel et al., 2001; Garnier et al., 2008; Pivard et al., 2008; Bailleul et al., 2016), Germany (Dietz-Pfeilstetter et al., 2006; Menzel, 2006; Elling et al., 2009; Middelhoff et al., 2009), the Netherlands (Tamis and de Jong, 2010), Great Britain (Crawley and Brown, 2004; Squire et al., 2011) and Austria (Pascher et al., 2006; 2010; 2016; 2017). Squire et al. (2011) show that regional presence and population size of OSR feral populations vary widely within Europe. In central France, the dynamics of feral populations were determined mainly by seed immigration from transport activities, for instance along paved roads, especially two-lane roads to silos (Pivard et al., 2008). It was estimated that 15% of establishments of feral OSR populations were due to seed spillage during transport activities. A study in central France identified seed dispersal through transportation networks to be the main cause for higher diversity of feral populations along roadsides than those along paths (Bailleul et al., 2016). In Tayside Scotland, United Kingdom, up to 60% of feral populations were estimated to be the result of transport losses (Charters et al., 1999). In addition, GM OSR seeds were identified in Switzerland, probably introduced as a contaminant of wheat imports (Schulze et al., 2015). Rostoks et al. (2019) and Sohn et al. (2021) specify possible routes for GM seeds to enter the European Union during import. In January 2023, certain European biofuel plants were shown to be sources of feral plants originating from contaminations with GM OSR seeds of a variety that is banned for cultivation in the European Union (https://www.reuters.com/article/france-rapeseed-gmo-idUSKBN2TZ1GP; accessed on 21 March 2023). In Manitoba, Canada, feral OSR along field edges generally resulted from seeding and management activities in local fields, whereas distribution patterns along roadsides were determined mostly by kernel transport occurring at the landscape or even at the regional scale (Knispel and McLachlan, 2010). Likewise, it was shown that seed losses during transport activities were the most likely cause of feral herbicide-tolerant OSR plants in southern Manitoba and in western Canada (Yoshimura et al., 2006).

Feral OSR populations containing traits of herbicide tolerance or point mutations promoting herbicide tolerance may serve as GM pollen sources for several years, contributing to the spread of traits of herbicide tolerance across the landscape. Herbicide tolerance may thus impair weed control at imported kernel loading sites, along railway lines, and transport roads. In Canada, GM herbicide tolerant OSR was approved for commercial cultivation in 1995. In western Canada this GM crop accounts for more than 95% of the OSR cultivation (Beckie and Warwick, 2010). The introgression of a glyphosate tolerance gene from OSR into its weedy relative *B. rapa* occurred in Canada under natural conditions (Warwick et al., 2008). In the US, where 90% of OSR fields are currently cultivated using GM OSR, herbicide-tolerant *B. napu*s was found at 45% (288 out of 634) of sample sites along highways and expressways, as well as around petrol stations and grocery stores (Schafer et al., 2011). In Japan, where GM OSR is not commercially grown, the occurrence of herbicide-tolerant OSR at ports of entry and along major roadways has been attributed to seed spillage from OSR imports (Saji et al., 2005; Kawata et al., 2009; Nishizawa et al., 2009; 2010; 2016). Aono et al. (2006) identified multiple herbicide tolerance traits in these feral OSR populations. In Switzerland—a country where neither cultivation nor import of GM OSR is allowed—GM glyphosate-resistant OSR (GT73) was identified in 2011 and 2012 in four out of 79 sample sites at railway stations and ports on the borders of France and Italy (Schoenenberger and D´Andrea, 2012; Hecht et al., 2013). Contaminated OSR kernel from freight trains is assumed to be the source of Swiss findings of GM OSR. The two most affected sites—the port at the Rhine River and the St. Johann freight railway station in Basel—were again monitored in 2013 and the presence of GT73 OSR was confirmed (Schulze et al., 2014). Consequently, import activities—transport, loading and handling—of GM OSR including genome-edited varieties are considered to be major activities leading to the establishment of feral GM OSR, even in countries without GM OSR cultivation. These activities are therefore a main concern in ecological risk assessment of GMOs in the European Union.

In the present study, we investigate the abundance and genetic diversity of feral OSR in Austria, considering conventional OSR as a model system to address questions associated with the entry and establishment of genome-edited OSR events in natural systems. In general, polymorphism, including microsatellites (simple sequence repeat: SSR), is used in variety identification—with ongoing ISO and UPOV standardization (International Organization for Standardization (ISO), 2015; ISO, 2019a; ISO, 2019b; UPOV, 2019a; UPOV, 2019b; UPOV, 2019c; UPOV, 2019d) and in processed products (Scarano et al., 2015) and could also be applied to determine the liability of the companies. Hence, in our study we examine SSR markers for characterising genetic diversity of commercial OSR varieties that have been cultivated for 10 years, as well as feral OSR plants sampled at import, loading and transhipment sites for kernels, as well as along subsequent transportation routes. We thus examine the hypothesis that genetic diversity will be higher along transportation routes (roads, railways, and ports (Pivard et al., 2008; Knispel and McLachlan, 2010; Schoenenberger and D´Andrea, 2012)) and especially at loading and handling sites of OSR compared to commercial varieties because of expected input of different OSR varieties from repeated seed spillage. Furthermore, we aim to identify genetic clusters to test for the possible presence of feral OSR gene pools not found in commercial varieties, potentially indicating the naturalization of imported OSR.

## 2 Materials and methods

### 2.1 Selection of sample sites

**FIGURE 1:**
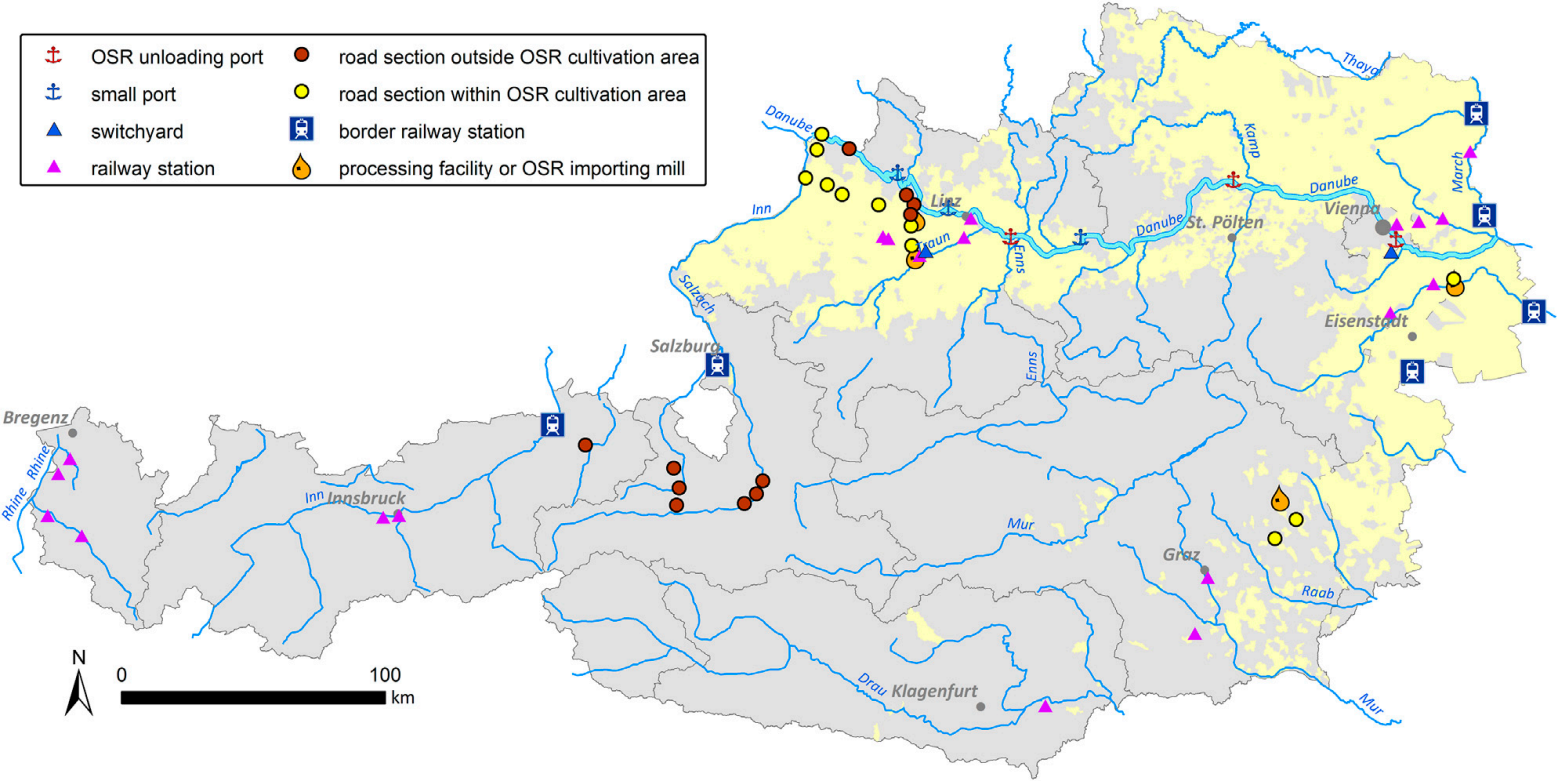
Location of the 60 selected sample sites all over Austria.

After interviewing 24 oil mills and processing facilities in Austria, four mills that import OSR from abroad were chosen for testing. Based on data from the Statistik Austria (http://www.statistik.at/; accessed on 17 March 2023) on the source countries of imported OSR for each Austrian Federal State (Bundesland), relevant sections of the Austrian railway transportation net, including railway stations, were selected. Similarly, the main road transport routes to importing oil mills were identified using route planner (Google Maps, Default settings). All data were imported into a Geographic Information System (ArcMap 10.2; ESRI, Redlands). Following Hecht et al. (2013), sampling concentrated on two main approaches, predefined hotspots and randomly selected sites (Figure 1). Predefined hotspots are defined as sample sites with a high expectation for spillage of imported kernels such as switchyards, border railway stations, main Danube ports, and OSR importing oil mills. A random sample of small river ports, small oil mills, and oilseed processing facilities was taken. Transport roads were divided into sections of 2 km length. These road sections as well as railway stations were randomly sampled inside OSR cultivation areas (based on IACS (Integrated Administration and Control System, https://agriculture.ec.europa.eu/common-agricultural-policy/financing-cap/assurance-and-audit/managing-payments_en) cultivation data of 2012 for Austrian municipalities) as well as outside OSR cultivation areas for reference purposes. Sections of the Danube River course, motorways and railway lines were not included, because of the exorbitant efforts for obtaining permits for this work, inadequate accessibility and/or security reasons (see Hecht et al., 2013). Using this procedure, altogether 60 sample sites were defined: border railway stations (RS-B: 6), switchyards (SY: 2), railway stations within OSR cultivation areas (RS-OSR: 10), railway stations outside OSR cultivation areas (RS-noOSR: 10), ports along the River Danube (PO: 6, 3 of these with OSR kernel loading referred to as main Danube ports), main transportation roads within OSR cultivation areas (RO-OSR: 11), main transportation roads outside OSR cultivation areas (RO-noOSR: 11), oil processing facilities (PF: 4; three OSR importing oil mills and one OSR processing company).

### 2.2 Sampling procedure of feral OSR plants

GPS and detailed OpenStreetMaps (OSM) were used to survey the 60 sample sites distributed throughout Austria (Figure 1). For sampling in the railway stations and switchyards, work permission was obtained, and railway safety training courses were attended. Plant sampling directly on the tracks was always accompanied by a railway station manager.

Young leaves—in some cases only very small leaves from exclusively seedlings of a feral population were available—of each feral OSR individual were collected, stored in a tea bag and dried with silica-gel. Additional information was recorded such as population size, sampling number of individuals, estimated age of the population, stage of maturity (blossoms, seeds, etc.), condition of plants, pest infestation as well as occurrence and frequency of hybridisation partners of OSR around the sample site. Altogether 2,113 feral OSR plants were sampled from 22 April to 19 August 2014 and again (on the same sites) from 23 April to 25 June 2015. Sampling was carried out for two consecutive years to verify the persistence of the feral populations studied and any increase or decrease in individual number within the populations. Separate analyses of the 2 years were not conducted due to large fluctuations—there was no evidence of feral plants at some sampling sites in one of the years.

### 2.3 Seed samples and DNA extraction

Seed samples of commercial OSR varieties were obtained from three of four Austrian seed breeders: Saatzucht Donau (http://www.saatzucht-donau.at; accessed on 14 April 2016), Raiffeisenware Austria (RWA; http://www.rwa.at; accessed on 14 April 2016), and KWS Austria Saat (http://www.kwsaustria.at; accessed on 14 April 2016). We have not obtained seed samples of OSR varieties from the company Pioneer Hi Bred Services (http://www.pioneer.com; accessed on 14 April 2016) which has a market-share of 1%–3% (pers. comm. with staff of Pioneer). Germination of seeds of the OSR varieties listed in Table 1 was performed at the Botanical Garden of the University of Vienna. Of those, five varieties Columbus, Contact, Honk, Mohican, and Vicking did not germinate at all (likely due to the age—approximately 17 or more years old—of the seed material already used in the previous study (Pascher et al., 2010)). For each of the remaining varieties, five individuals were harvested and dried in silica-gel except for the varieties Caracas, Henry, Tenno and Jolly, where due to poor germination only three individuals each were available for testing.

**TABLE 1:** Oilseed rape varieties grown in Austria between 2005 and 2015 and varieties imported from EU countries.

Name of variety	Applicant in Austria	Breeder^a^	Year of seed harvest	Type^b^	List of varieties^c^
Adriana	RWA	F	2013	OP	BSL
Alabaster	Saatzucht Donau	Limagrain	2013	HY	BSL
Albatros	Saatzucht Donau	Limagrain, F	2013	HY	BSL
Ametyst	RWA	S	2013	OP	BSL
Artoga	Saatzucht Donau	Limagrain, F	2013	HY	BSL
Californium	Saatzucht Donau	Monsanto	possibly 2006	OP	BSL
Caracas	Saatzucht Donau	Monsanto	possibly 2005	OP	BSL
Carousel	Saatzucht Donau	Monsanto	possibly 2002	OP	BSL
Casoar	Saatzucht Donau	Monsanto, United States of America	2013	OP	BSL
Castille	Saatzucht Donau	Monsanto, United States of America	possibly 2005	OP	BSL
Columbus	Saatzucht Donau	Monsanto	possibly 2002	OP	BSL
Contact	Saatzucht Donau	Monsanto	2005	OP	BSL
Digger	KWS				BSL
DK Excellium	Saatzucht Donau	Monsanto, United States of America	2013	HY	BSL
DK Exfield	RWA	Monsanto, United States of America	2013	HY	BSL
DK Expertise	Saatzucht Donau	Monsanto, United States of America	2013	HY	BSL
DK Expower	Saatzucht Donau	Monsanto, United States of America	2013	HY	BSL
DK Exstorm	RWA	Monsanto, United States of America	2013		BSL
DK Sequoia	RWA	Monsanto, United States of America	2013		BSL
ES Solist	RWA		2013		BSL
Gloria	Saatzucht Donau	Syngenta, CH	2013	OP	BSL
Graf	Saatzucht Donau	Monsanto, United States of America	2013	HY	BSL
Harry	Saatzucht Donau	SZD, A	2010	OP	BSL
Henry	Saatzucht Donau	SZD, A	2013	OP	BSL
Honk	Saatzucht Donau	Groenbroek	unknown	OP	BSL
Kutiba	RWA		2011		BSL
Landoga	Saatzucht Donau	Limagrain	2013	OP	BSL
Mickey	Saatzucht Donau	SZD, A	2007	OP	BSL
Mohican	Saatzucht Donau	CPB	unknown	OP	BSL
NK Petrol	Saatzucht Donau	Syngenta	2013	HY	BSL
Peter 29	RWA		2013		BSL
Remy	KWS	D		OP	BSL
Sammy	Saatzucht Donau	SZD, A	2008	OP	BSL
Sherlock	KWS	D		OP	BSL
Sherpa	RWA	D	2013		BSL
Sidney	Saatzucht Donau	SZD	2011	OP	BSL
Tenno	Saatzucht Donau	NPZ	possibly 2006	HY	BSL
Viking	Saatzucht Donau	NPZ	possibly 2002	HY	BSL
WKR Janus	RWA		2012		BSL
DK Sedona	Saatzucht Donau	Monsanto	2010	HY	EU
Freddy	Saatzucht Donau	SZD	2013	OP	EU
Jimmy	Saatzucht Donau	SZD	2007	OP	EU
Jolly	Saatzucht Donau	SZD	2011	OP	EU
Lenny	Saatzucht Donau	SZD	2011	OP	EU
Orlando	Saatzucht Donau	Monsanto/SZD	2008	HY	EU
Pedro	Saatzucht Donau	Monsanto/SZD	2008	HY	EU
Ricky	Saatzucht Donau	SZD	2013	OP	EU
Tommy	Saatzucht Donau	SZD	2012	OP	EU

^a^ CPB, former name of KWS United Kingdom, Ltd.; SZD, Saatzucht Donau; NPZ, Norddeutsche Pflanzenzucht.

^b^ HY, hybrid; OP, open pollinators (= variety lines).

^c^ BSL, Österreichische Beschreibende Sortenliste (Austrian Variety Database); EU, European Union.

DNA from all 2,113 feral OSR plants and 217 individuals from 45 OSR varieties, amounting to a total of 2,330 individuals (excluding replicate extractions), was extracted from 10 mg dried plant material using the Dneasy 96 Plant Kit (Qiagen, Hilden, Germany) following the manufacturer’s instructions. For each extraction plate, comprising 96 samples, on average three randomly chosen extraction replicates were included. Several of the feral plants dropped out during analysis because they could not be extracted due to low amount of leaf-material of sampled seedlings or insufficient quality due to pest infested leaves or old seed material. The overall number of samples with which amplification was attempted was 2,116 (report of Pascher et al., 2016).

### 2.4 Genetic analysis

Seven SSR primers (Na12-A08, Na12-C06, Na-C08, Na12-C12, Na12-D11, Na12-E01 (amplifying two loci termed Na12-E01a and Na12-E01b), Na12-E06A) that amplify eight microsatellite loci have been used successfully in previous studies (Pascher et al., 2006; Pascher et al., 2010). To make full use of the dyes, an eighth primer pair was added. Based on Lowe et al. (2004) six primer pairs (Na10-B04, Na10-C01, Na10-C06, Na10-G06, Na12-H09, Na14-C12) were chosen out of 31 that were polymorphic in *B. napus*. Of those, Na10-C01 amplified all tested samples successfully and was therefore included in the analysis. PCR amplifications were conducted in two fourfold multiplexed reactions (primer labelling given in parentheses): the first reaction included Na12-A08 (6-FAM), Na12-C08 (VIC), Na12-C12 (NED), Na12-D11 (PET); the second reaction consisted of Na12-C06 (6-FAM), Na12-E01 (VIC), Na12-E06A (NED) and Na10-C01 (PET). All labelled primers were obtained from Thermo Fisher Scientific (Waltham, United States). PCR amplifications were performed on a GeneAmp PCR System 9700 Thermocycler (Applied Biosystems, Foster City, United States) using the following PCR program (recommended for the used PCR kit): denaturation at 95°C for 5 min, followed by 24 cycles each with 95°C for 30 s, 60°C for 90 s and 72°C for 30 s, followed by a final elongation step of 30 min at 60°C. The PCR reaction mix of 11.5 µL contained 6 µL of Type-it Microsatellite PCR Kit (Qiagen, Hilden, Germany), 0.2 µL of each primer (10 µM), 1.5 µL of template DNA of unknown concentration, and 2.4 µL double-distilled water. The products of the two PCR reactions were purified separately using Sephadex (GE Healthcare Bio-Sciences AB, Uppsala, Sweden), mixed 1:1 and separated on a capillary sequencer ABI 3130xl (Applied Biosystems) using GeneScan 600 LIZ (Thermo Fischer Scientific) as internal size standard in accordance with the manufacturer’s instructions.

Fragments were sized and manually scored using GeneScan 3.7 and Genotyper 3.7 (both Applied Biosystems). Assignment of alleles of equally labelled loci (e.g., Na12-C08 and Na12-E01) was based on information on allele size range available from previous studies (Pascher et al., 2000; Pascher et al., 2006). For primer pair Na10-C01, no literature values for scoring were available. As this primer consistently amplified two distinct loci, that showed independent patterns of variation, both were scored (henceforth listed as Na10-C01a and Na10-C01b) and included in the matrix, which eventually contained 2,185 samples. Replicates resulted, as expected, in profiles congruent with those of the original samples—four replicates failed to amplify completely or nearly so—and were removed, resulting in a matrix of 2,116 samples.

This raw data matrix was further processed as follows. Samples that did not amplify for more than one locus (not considering Na12-E01b; see Results) even after the second attempt were removed (38 samples), reducing the data matrix to 2,078 samples. Alleles which did not fit into the expected step-size range (all amplified loci have two-base pair motifs (Lowe et al., 2004)) were re-coded as missing data: one each in both loci of Na10-C01; two each in Na12-C06, Na12-E01a and Na12-C08 and 13 in Na12-E06a (21 alleles in 20 samples). Likewise, loci with more than two alleles per individual were coded as missing data, ignoring alleles that in the previous step had already been coded as missing data (one case each for loci Na12-E01a, Na12-C08 (in the same individual), and Na12-E06a), affecting five times Na12-A08, ten times locus Na12-C08 and 15 times locus Na12-E06a. Three samples that after this re-coding had two loci (again not considering Na12-E01b) with missing data were removed, resulting in a final matrix containing 2,075 samples (report of Pascher et al., 2016).

### 2.5 Statistical analyses

Data descriptors and diversity statistics (both for loci, populations, and population groups) were calculated using GenAlEx 6.5 (Peakall and Smouse, 2006; Peakall and Smouse, 2012) and—for allelic richness only—Fstat 2.9.3.2 (Goudet, 1995; Goudet, 2001). As these programs cannot handle mixtures of missing data and allele sizes within the same locus of a sample, in four cases (one each in Na12-E01a and Na12-C08 and twice in Na12-C06) the observed allele was re-coded as missing data. Diversity measures were compared using Mann-Whitney tests or Kruskal–Wallis tests using PAST 3.09 (Hammer et al., 2001), where *p*-values were estimated using 9,999 Monte Carlo permutations. Differentiation among six pre-defined population groups (RS, comprising RS-OSR and RS-noOSR: railway stations; SY: switchyards; RS-B: border railway stations; PO: ports (main Danube ports and small Danube ports); RO, comprising RO-OSR and RO-noOSR: road sections; MI: oil mills and companies) was quantified using a three-level AMOVA (populations with fewer than ten individuals were removed: RS-noOSR1, RS-noOSR4, RS-noOSR6, RS-OSR10, RS-B3, RS-B7, MI2, RO-OSR9, all commercial varieties) in Arlequin 3.5 (Excoffier and Lischer, 2010); significance of F_ST_ values was estimated using 1,000 permutations. Pairwise F_ST_ values, using number of different alleles as distance method (Weir and Cockerham, 1984; Michalakis and Excoffier, 1996), between seven population groups (as for AMOVA, but including the entirety of commercial varieties as a seventh group) were calculated using Arlequin 3.5; significance of F_ST_ values was assessed using 1,000 permutations. Allelic richness accumulation curves were estimated for each of the seven population groups using the R-package ARES 1.2-2 (van Loon et al., 2007) run on R 2.4.1 for Windows (R Development Core Team 2008; available from https://cran.r-project.org/) with a maximum of 1,000 individuals (thus, at least twice the size of a given population group) and 500 bootstrap replicates to obtain confidence intervals. As marker Na12-E01b had 51.57% missing data (see Results), all estimates were calculated both using the complete data set of ten loci and a reduced data set of nine loci (i.e., without Na12-E01b). Arlequin by default removes loci with more than 5% missing data; consequently, calculations of AMOVA and pairwise F_ST_ values are based on the reduced data set.

Population structure of the entire data set (feral populations plus commercial varieties) was inferred using the non-hierarchical Bayesian clustering method implemented in Structure 2.3.4 (Pritchard et al., 2000; Falush et al., 2003; Falush et al., 2007) assuming admixture and correlated allele frequencies. The admixture model allows that individuals may have—but do not need to have—mixed ancestry; the correlated allele frequency model allows allele frequencies between inferred populations to be quite similar, which tends to improve clustering for closely related populations (Falush et al., 2003). For each number of clusters *(K)*, ranging from *K* = 1 to *K* = 20, ten independent runs were performed using a burn-in of 2×10^5^ iterations followed by 2×10^6^ additional MCMC iterations for sampling. For detecting the number of clusters, we used the DeltaK statistic (Evanno et al., 2005), calculated using Structure Harvester Web 0.6.94 (Earl and von Holdt, 2012). The cluster output from Structure was aligned using Clumpp 1.1.1 (Jakobsson and Rosenberg, 2007) and visualized using Distruct 1.1 (Rosenberg, 2004) (report of Pascher et al., 2016).

## 3 Results

### 3.1 Occurrence of feral OSR and its hybridisation partners in sample sites

**FIGURE 2:**
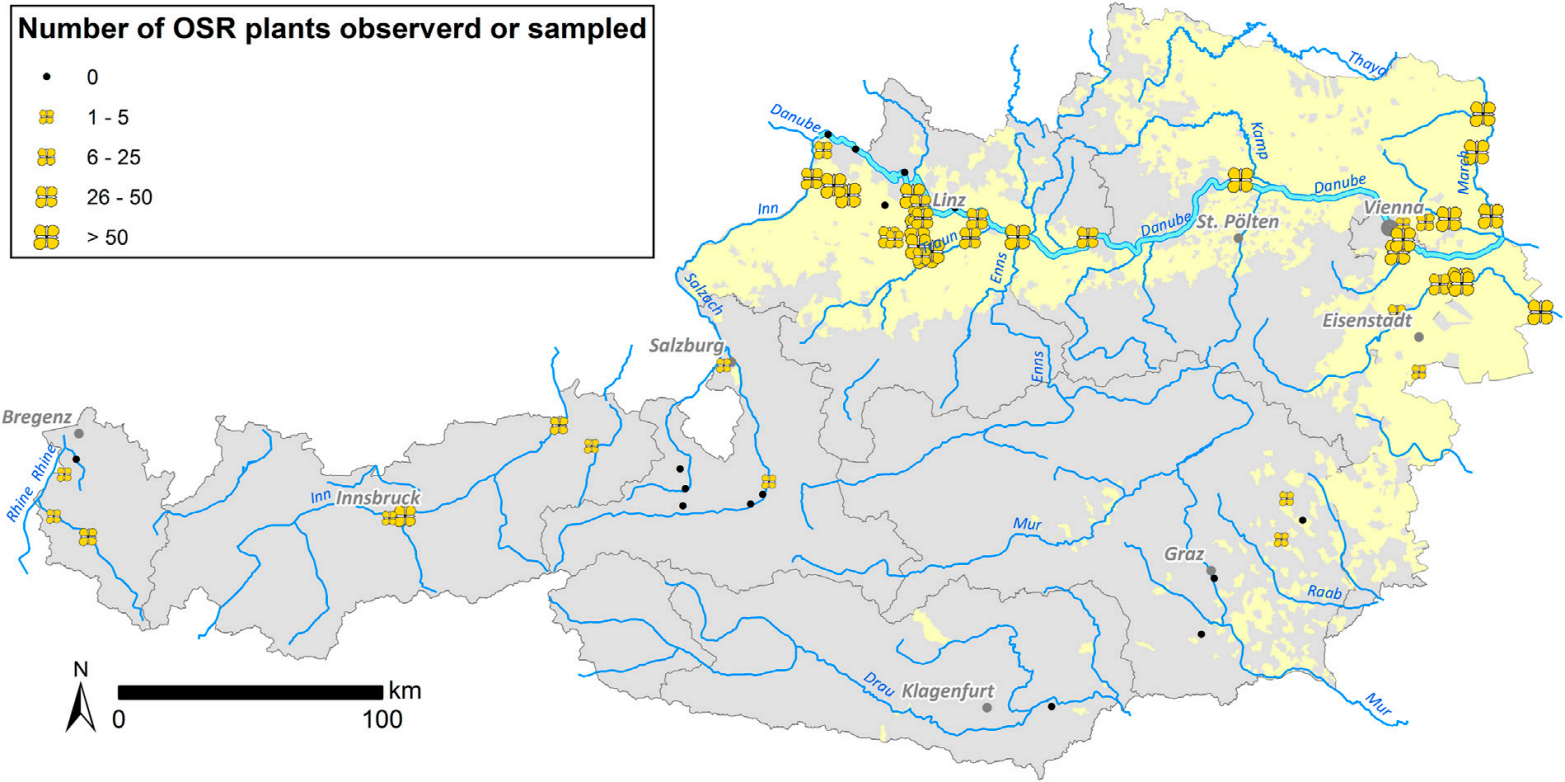
Sizes of feral OSR populations observed/sampled at the sample sites summarized for 2014 and 2015. Occurrence of feral OSR is indicated with yellow flowers—flower sizes according to individual numbers—in the figure. The Austrian OSR cultivation areas are marked in light yellow.

In 2014 and 2015, a total of 2,113 feral plant individuals were collected for testing (Table 2). Feral OSR was found at 44 of the 60 sites surveyed (Figure 2). Most of these 44 sites were situated within OSR cropping areas. With few exceptions, no feral OSR plants were found along roads through areas without OSR cultivation and probably without OSR transportation. Nearly all observed plants were in good condition, flowering and producing seeds. Growing sites of OSR showed a wide ecological habitat-spectrum within disturbed habitats such as port sites, roadsides, railway tracks, ruderal sites, and riverbanks. Sizes of feral populations differed strongly, ranging from one to estimated more than 1,400 plants on 2 km road section and with several hundred plants at the area of the largest Austrian oil mill (Bunge), resulting in a median of 30 individuals per site. Generally, large population sizes were observed at sites where OSR—also imported kernels—is loaded and handled.

**TABLE 2:** Sample sites and number of sampled feral oilseed rape plants at location in 2014 and 2015.

Sampling type	Sampling site	Federal state	Sampled individuals	Sampled individuals
**Railway stations outside (BHK) and within (BHR) Austrian OSR cultivation areas**			2014	2015		
RS-noOSR1	Völs	Tyrol	3	0		
RS-noOSR2	Linz - Wahringerbahnhof	Upper Austria	40	0		
RS-noOSR3	Innsbruck	Tyrol	0	28		
RS-noOSR4	Hohenems	Vorarlberg	0	1		
RS-noOSR5	Graz Ostbahnhof	Styria	0	0		
RS-noOSR6	Frastanz	Vorarlberg	0	1		
RS-noOSR7	Preding-Wieselsdorf	Styria	0	0		
RS-noOSR8	Bludenz	Vorarlberg	0	11		
RS-noOSR9	Dornbirn	Vorarlberg	0	0		
RS-noOSR10	Völkermarkt-Kühnsdorf	Carinthia	0	0		
RS-OSR1	Wels-Hauptbahnhof	Upper Austria	26	47		
RS-OSR2	Schlüsslberg	Upper Austria	11	3		
RS-OSR3	Raasdorf	Lower Austria	5	18		
RS-OSR4	Siebenbrunn-Leopoldsdorf	Lower Austria	26	29		
RS-OSR5	Grieskirchen	Upper Austria	21	28		
RS-OSR6	Trautmannsdorf a. d. Leitha	Burgenland	17	11		
RS-OSR7	Wampersdorf	Lower Austria	1	10		
RS-OSR8	Traun	Upper Austria	1	25		
RS-OSR9	Dürnkrut	Lower Austria	53	38		
RS-OSR10	Hirschstetten-Aspern	Lower Austria	0	1		
**Ports**						
PO1	Hafen Albern	Vienna/Lower Austria	35	61		
PO2	Krems	Lower Austria	55	64		
PO3	Enns	Upper Austria	30	50		
PO4	Grein	Upper Austria	20	22		
PO5	Obermühl an der Donau	Upper Austria	0	0		
PO6	Wilhering	Upper Austria	0	0		
**Border railway stations**						
RS-B1	Nickelsdorf	Lower Austria	24	50		
RS-B2	Hohenau	Lower Austria	50	49		
RS-B3	Baumgarten	Burgenland	0	2		
RS-B4	Kufstein	Tyrol	2	18		
RS-B5	Marchegg	Lower Austria	40	43		
RS-B7	Salzburg	Salzburg	1	2		
**Switchyards**						
SY1	Kledering Verschubbahnhof	Lower Austria	98	60		
SY2	Wels Verschubbahnhof	Upper Austria	21	47		
**Road sections within the Austrian OSR cultivation area**			2014	2015		
RO-OSR1	Katzdorf bei Wallern a. d. Trattnach	Upper Austria	30	34		
RO-OSR2	Aumühle bei Wels	Upper Austria	30	50		
RO-OSR3	Thall bei Waizenkirchen	Upper Austria				
RO-OSR4	Taufkirchen- Leoprechting	Upper Austria	20	40		
RO-OSR5	Thalmannsbach	Upper Austria	20	40		
RO-OSR6	St. Florian am Inn	Upper Austria	12	15		
RO-OSR7	Hinding (an der Donau)	Upper Austria	0	0		
RO-OSR8	Schardenberg-Steinbrunn	Upper Austria	2	15		
RO-OSR9	Pischelsdorf in der Steiermark	Styria	3	1		
RO-OSR10	Dienersdorf-Kaindorf	Styria	0	0		
RO-OSR11	Bruck an der Leitha	Lower Austria	49	58		
**Road sections outside of the Austrian OSR cultivation area**						
RO-noOSR1	Eferding	Upper Austria	22	1		
RO-noOSR2	Hilkering bei Aschach an der Donau	Upper Austria	8	45		
RO-noOSR3	Pupping und Karling bei Aschach	Upper Austria	26	3		
RO-noOSR4	Engelhartszell (an der Donau)	Upper Austria	0	0		
RO-noOSR5	St. Johann im Pongau	Salzburg	0	0		
RO-noOSR6	Bischofshofen	Salzburg	0	4		
RO-noOSR7	Zell am See	Salzburg	0	0		
RO-noOSR8	Maishofen	Salzburg	0	0		
RO-noOSR9	Schwarzach im Ponau	Salzburg	0	0		
RO-noOSR10	Saalfelden am Steinernen Meer	Salzburg	0	0		
RO-noOSR11	Going (beim Wilden Kaiser)	Tyrol	0	0		
**Oil processing facilities**						
PF1	Vereinigte Fettwaren - Wels	Upper Austria	21	0		
PF2	Fandler - Pöllau	Styria	0	2		
PF3	Bunge - Bruck an der Leitha	Burgenland	165	60		
PF4	Raab - Fraham	Upper Austria	13	25		
		**Samples**	**1001**	**1112**		

In Table bold values are just used as a typographic way to distinguish the row showing the means from the those showing other values

At several of the 60 sample sites eleven potentially cross-breeding species were registered (Supplementary Table S1). *Sinapis arvensis* was the most common possible hybridisation partner, with 21 records (Pascher et al., 2016), while *Diplotaxis tenuifolia* was the second most frequently observed species (20 records). In case of *D. tenuifolia*, successful hybridisation with OSR has already been proven under field conditions (Chevré et al., 2004). As perennial wall-rocket is also cultivated as a crop, the observed hybrids would also pose a challenge for the coexistence of contaminated wild weeds with the cultivated crop (Hall et al., 2012; Caruso et al., 2018). At each of 25 sample sites, two potential cross breeding species of OSR were recorded, while at one sample site even five species were present.

### 3.2 SSR (simple sequence repeats) analyses

Locus-by-locus descriptive statistics for commercial varieties *versus* feral populations are presented in Supplementary Table S2. Although all indices except the normalized Shannon Diversity Index, *I*_nor_, suggested that feral populations had higher genetic diversity than the commercial varieties, none of these differences were statistically significant (*p*-values for the reduced data set given in parentheses): effective number of alleles, *n*_e_: *p* = 0.636 (0.730); observed heterozygosity, *H*_O_: *p* = 0.986 (1); expected heterozygosity, *H*_E_: *p* = 0.636 (0.733); normalized Shannon Index, *I*_nor_: *p* = 0.591 (0.559); allelic richness, AR: *p* = 0.142 (0.166).

When considering multi-locus genotypes jointly for all feral populations, they had higher levels of genetic diversity than commercial varieties (Table 3) which were statistically highly significant (*p* < 0.001) except for observed heterozygosity (*p* = 0.108 and *p* = 0.124 for the complete and the reduced data set, respectively); this pattern stayed the same when only considering populations (varieties) with at least four individuals (data not shown). When considering multi-locus genotypes for population groups separately (railway stations, switchyards, border railway stations, ports, road sections, oil processing facilities: oil mills and OSR processing companies), these showed different levels of genetic diversity, but the rank of each group differed between diversity measures (and occasionally also between the complete and the reduced data sets). For instance, switchyards had the highest percentage of polymorphic loci, but only medium levels of observed heterozygosity. Differences in genetic diversity were statistically not significant (*p*-values for the reduced data set given in parentheses): effective number of alleles, *n*_e_: *p* = 0.355 (0.246); percentage of polymorphic loci, Per_poly_: *p* = 0.468 (0.471); expected heterozygosity, *H*_E_: *p* = 0.375 (0.222); normalized Shannon Index, *I*_nor_: *p* = 0.302 (0.118). The only exception was observed heterozygosity, *H*_O_, which was significant for the complete but not the reduced data set: *p* = 0.038 (0.079). Although in pairwise Mann-Whitney tests several cases of significant differences were found (e.g., railway stations *versus* ports and *versus* road sections), these became non-significant after sequential Bonferroni correction (report of Pascher et al., 2016). The number of private alleles (i.e., alleles exclusively present in one group) was 8 out of 95 (8.4%) for railway stations, 1 out of 74 (1.4%) for switchyards, 2 out of 69 (2.9%) for border railway stations, 10 out of 91 (11%) for ports (the 3 main Danube ports), 7 out of 90 (7.8%) for road sections, and 5 out of 83 (6%) for oil processing facilities. Comparing feral populations with varieties, in the former 70 out of 134 alleles (52.2%) were private alleles, whereas in the latter only 1 out of 65 alleles (1.5%) was a private allele (see Supplementary Table S2 for these numbers separated by locus). Frequencies of private alleles were low when treating categories of sampling sites separately (railway stations: 1.37%; switchyards: 0.23%; border railway stations: 0.39%; ports: 2.85%; road sections: 1.18%; oil processing facilities: 1.2%), but rose to 18.19% when treating all feral populations jointly. In contrast, the frequency of private alleles in varieties was low (0.47%).

**TABLE 3:** Descriptive genetic statistics for feral populations.

	Sample size	Descriptive statistics^a^ ^,^ ^b^							
		Avg. No. Data/Locus^a^	*N*_GT_	*n*_a_	*n*_e_	Per_poly_	*H*_O_	*H*_E_	*I*_nor_
**Feral Populations** **Railway Stations**									
RS-noOSR1	3	3.00 (3.00)	3 (3)	1.400 (1.444)	1.340 (1.378)	40.00 (44.44)	0.167 (0.185)	0.220 (0.244)	0.237 (0.263)
RS-noOSR2	36	34.30 (35.78)	34 (34)	4.200 (4.444)	1.848 (1.919)	100.00 (100.00)	0.145 (0.161)	0.367 (0.388)	0.203 (0.214)
RS-noOSR3	27	25.60 (26.67)	26 (26)	3.600 (3.667)	2.067 (2.071)	100.00 (100.00)	0.223 (0.247)	0.488 (0.484)	0.262 (0.258)
RS-noOSR4	1	1.00 (1.00)	1 (1)	1.100 (1.111)	1.100 (1.111)	10.00 (11.11)	0.100 (0.111)	0.100 (0.111)	0.000 (0.000)
RS-noOSR6	1	1.00 (1.00)	1 (1)	1.200 (1.111)	1.200 (1.111)	20.00 (11.11)	0.200 (0.111)	0.200 (0.111)	0.000 (0.000)
RS-noOSR8	10	9.40 (9.89)	10 (10)	2.700 (2.889)	1.817 (1.908)	70.00 (77.78)	0.172 (0.191)	0.358 (0.398)	0.268 (0.297)
RS-OSR1	68	63.10 (67.56)	67 (67)	6.300 (6.556)	2.515 (2.582)	100.00 (100.00)	0.177 (0.187)	0.466 (0.464)	0.241 (0.235)
RS-OSR10	1	0.90 (1.00)	1 (1)	1.100 (1.222)	1.100 (1.222)	20.00 (22.22)	0.200 (0.222)	0.200 (0.222)	0.000 (0.000)
RS-OSR2	12	11.00 (11.56)	12 (12)	3.400 (3.444)	2.224 (2.175)	90.00 (88.89)	0.148 (0.146)	0.491 (0.470)	0.368 (0.345)
RS-OSR3	23	21.50 (23.00)	19 (19)	3.200 (3.333)	2.108 (2.133)	90.00 (88.89)	0.222 (0.246)	0.427 (0.419)	0.247 (0.239)
RS-OSR4	56	52.10 (55.89)	40 (39)	4.700 (4.778)	1.958 (1.877)	100.00 (100.00)	0.110 (0.103)	0.383 (0.353)	0.201 (0.179)
RS-OSR5	48	44.70 (47.00)	47 (45)	5.400 (5.333)	2.214 (2.204)	100.00 (100.00)	0.148 (0.159)	0.447 (0.432)	0.240 (0.228)
RS-OSR6	28	26.60 (27.78)	28 (28)	4.800 (4.556)	2.540 (2.401)	90.00 (88.89)	0.209 (0.218)	0.459 (0.425)	0.291 (0.259)
RS-OSR7	11	10.40 (11.00)	11 (11)	2.700 (2.667)	1.951 (1.915)	70.00 (66.67)	0.182 (0.202)	0.381 (0.354)	0.290 (0.257)
RS-OSR8	26	23.70 (26.00)	26 (26)	3.500 (3.778)	1.976 (2.084)	80.00 (88.89)	0.150 (0.167)	0.354 (0.394)	0.211 (0.234)
RS-OSR9	53	50.20 (52.89)	51 (50)	3.600 (3.667)	1.998 (1.998)	80.00 (77.78)	0.191 (0.212)	0.384 (0.370)	0.179 (0.172)
*Mean* *^c^*	*25.3 (33.2)*	*23.66 (25.06) [31.05 (32.92)]*	*23.56 (23.31) [30.92 (30.58)]*	*3.306 (3.375) [4.008 (4.093)]*	*1.872 (1.881) [2.101 (2.106)]*	*72.50 (72.92) [89.17 (89.82)]*	*0.171 (0.179) [0.173 (0.187)]*	*0.358 (0.352) [0.417 (0.413)]*	*0.249 (0.245) [0.250 (0.245)]*
**Switchyards**									
SY1	155	147.50 (153.22)	131 (125)	6.500 (6.333)	2.401 (2.419)	100.00 (100.00)	0.183 (0.188)	0.429 (0.415)	0.181 (0.175)
SY2	68	64.10 (67.11)	65 (64)	5.400 (5.444)	2.395 (2.387)	100.00 (100.00)	0.148 (0.153)	0.456 (0.440)	0.220 (0.211)
*Mean*	*111.5*	*105.80 (110.17)*	*98.00 (94.50)*	*5.950 (5.889)*	*2.398 (2.403)*	*100.00 (100.00)*	*0.166 (0.171)*	*0.443 (0.427)*	*0.201 (0.193)*
**Border Railway Stations**									
RS-B1	71	67.30 (70.56)	71 (71)	5.200 (5.222)	2.539 (2.547)	100.00 (100.00)	0.170 (0.178)	0.478 (0.464)	0.228 (0.221)
RS-B2	95	87.70 (94.44)	90 (89)	5.800 (5.889)	2.412 (2.501)	100.00 (100.00)	0.200 (0.214)	0.430 (0.435)	0.207 (0.203)
RS-B3	3	2.70 (2.78)	2 (2)	1.500 (1.556)	1.457 (1.508)	40.00 (44.44)	0.400 (0.444)	0.293 (0.326)	0.242 (0.273)
RS-B4	19	17.20 (18.78)	11 (11)	2.800 (3.000)	1.525 (1.583)	90.00 (100.00)	0.112 (0.125)	0.285 (0.317)	0.176 (0.196)
RS-B5	68	64.40 (67.89)	65 (63)	4.400 (4.333)	2.342 (2.241)	90.00 (88.89)	0.133 (0.144)	0.445 (0.417)	0.209 (0.190)
RS-B7	3	2.70 (2.89)	3 (3)	1.700 (1.778)	1.454 (1.505)	60.00 (66.67)	0.150 (0.167)	0.297 (0.330)	0.412 (0.412)
*Mean* *^c^*	*43.2 (63.3)*	*40.33 (42.89) [59.15 (62.92)]*	*40.33 (39.83) [59.25 (58.50)]*	*3.567 (3.630) [4.550 (4.611)]*	*1.955 (1.981) [2.205 (2.218)]*	*80.00 (83.33) [95.00 (97.22)]*	*0.194 (0.212) [0.154 (0.165)]*	*0.371 (0.381) [0.410 (0.408)]*	*0.246 (0.249) [0.205 (0.249)]*
**Ports**									
PO1	87	81.50 (85.89)	85 (81)	6.300 (6.444)	2.386 (2.361)	100.00 (100.00)	0.150 (0.159)	0.452 (0.432)	0.215 (0.206)
PO2	114	107.80 (113.11)	111 (106)	6.300 (6.333)	2.313 (2.252)	100.00 (100.00)	0.135 (0.144)	0.454 (0.432)	0.198 (0.186)
PO3	60	55.80 (59.00)	58 (56)	5.200 (5.333)	2.333 (2.240)	90.00 (88.89)	0.127 (0.137)	0.448 (0.420)	0.224 (0.207)
PO4	39	37.60 (38.89)	32 (28)	3.400 (3.111)	1.865 (1.784)	70.00 (66.67)	0.081 (0.085)	0.324 (0.290)	0.175 (0.151)
*Mean*	*75.0*	*70.68 (74.22)*	*71.50 (67.75)*	*5.300 (5.306)*	*2.225 (2.159)*	*90.00 (88.89)*	*0.123 (0.131)*	*0.419 (0.394)*	*0.203 (0.187)*
**Road sections**									
RO-noOSR1	23	21.60 (22.89)	23 (23)	4.300 (4.333)	2.086 (1.965)	100.00 (100.00)	0.164 (0.171)	0.446 (0.416)	0.289 (0.260)
RO-noOSR2	52	49.50 (51.89)	46 (39)	3.900 (3.667)	1.757 (1.632)	80.00 (77.78)	0.110 (0.122)	0.298 (0.257)	0.157 (0.130)
RO-noOSR3	18	17.10 (18.00)	17 (14)	3.100 (3.111)	1.518 (1.467)	90.00 (88.89)	0.122 (0.136)	0.303 (0.279)	0.199 (0.178)
RO-OSR11	84	78.60 (82.67)	80 (78)	5.700 (5.556)	2.309 (2.305)	100.00 (100.00)	0.138 (0.150)	0.438 (0.422)	0.207 (0.195)
RO-OSR1	55	52.30 (54.89)	45 (40)	3.800 (3.778)	1.803 (1.729)	90.00 (88.89)	0.078 (0.079)	0.342 (0.313)	0.169 (0.151)
RO-OSR2	67	63.50 (66.89)	59 (52)	4.900 (4.556)	2.022 (1.872)	100.00 (100.00)	0.097 (0.105)	0.389 (0.352)	0.194 (0.167)
RO-OSR4	59	56.00 (58.56)	54 (53)	4.800 (4.667)	2.194 (2.106)	100.00 (100.00)	0.111 (0.124)	0.437 (0.411)	0.221 (0.203)
RO-OSR5	59	55.30 (58.33)	55 (51)	5.600 (5.556)	2.404 (2.245)	100.00 (100.00)	0.137 (0.152)	0.441 (0.406)	0.229 (0.204)
RO-OSR6	27	25.70 (26.78)	26 (26)	3.600 (3.556)	2.056 (2.000)	90.00 (88.89)	0.190 (0.211)	0.419 (0.395)	0.239 (0.223)
RO-OSR8	17	15.50 (16.89)	16 (16)	2.700 (2.778)	1.940 (1.955)	70.00 (66.67)	0.210 (0.233)	0.369 (0.351)	0.257 (0.222)
RO-OSR9	3	3.00 (3.00)	3 (3)	1.600 (1.667)	1.477 (1.530)	50.00 (55.56)	0.033 (0.037)	0.287 (0.319)	0.324 (0.360)
*Mean* *^c^*	*42.2 (46.1)*	*39.83 (41.89) [43.51 (45.78)]*	*38.55 (35.91) [42.10 (39.20)]*	*4.000 (3.929) [4.240 (4.156)]*	*1.961 (1.891) [2.009 (1.928)]*	*88.18 (87.88) [92.000 (91.112)]*	*0.126 (0.138) [0.136 (0.148)]*	*0.379 (0.356) [0.388 (0.360)]*	*0.226 (0.208) [0.216 (0.208)]*
**Oil Processing Facilities**									
PF1	21	19.30 (20.89)	21 (21)	3.200 (3.333)	1.805 (1.842)	100.00 (100.00)	0.179 (0.198)	0.397 (0.401)	0.242 (0.234)
PF2	3	2.60 (2.89)	3 (3)	2.200 (2.444)	1.921 (2.135)	80.00 (88.89)	0.233 (0.259)	0.487 (0.541)	0.699 (0.699)
PF3	151	141.60 (150.11)	141 (136)	7.200 (7.222)	2.310 (2.306)	100.00 (100.00)	0.169 (0.177)	0.435 (0.420)	0.188 (0.179)
PF4	33	31.10 (32.78)	32 (32)	4.300 (4.333)	2.182 (2.122)	100.00 (100.00)	0.193 (0.186)	0.464 (0.443)	0.255 (0.238)
*Mean* *^c^*	*52.0 (68.3)*	*48.65 (51.67) (64.00 (67.93))*	*49.25 (48.00) (64.67 (63.00))*	*4.225 (4.333) (4.900 (4.963))*	*2.055 (2.101) (2.099 (2.090))*	*95.00 (97.22) (100.00 (100.00))*	*0.193 (0.205) (0.180 (0.187))*	*0.446 (0.451) (0.432 (0.421))*	*0.346 (0.337) (0.228 (0.217))*
*Grand Mean* *^c^*	*43.2 (52.6)*	*40.64 (42.86) [49.45 (52.16)]*	*40.05 (38.58) [48.71 (46.91)]*	*3.914 (3.938) [4.471 (4.486)]*	*1.981 (1.968) [2.118 (2.089)]*	*82.56 (83.20) [92.29 (92.38)]*	*0.160 (0.171) [0.155 (0.166)]*	*0.383 (0.374) [0.411 (0.397)]*	*0.245 (0.236) [0.225 (0.213)]*
**Commercial Varieties**									
*Mean* *^c^*	*4.8 (5.0)*	*4.57 (9.58) [4.69 (9.84)]*	*2.67 (2.67) [2.71 (2.71)]*	*1.362 (1.420) [1.374 (1.431)]*	*1.240 (1.290) [1.247 (1.296)]*	*30.00 (31.61) [30.71 (32.28)]*	*0.148 (0.161) [0.150 (0.162)]*	*0.152 (0.159) [0.154 (0.162)]*	*0.137 (0.141) [0.137 (0.141)]*

^a^ Avg. No. Data/Locus = sample size as average number of data per locus; N_GT_, number of genotypes; *n*_a_ = observed number of alleles; *n*_e_ = effective number of alleles (Brown and Weir, 1983); Per_poly_ = percentage of polymorphic loci; *H*_O_, observed heterozygosity; *H*_E_, unbiased expected heterozygosity (Nei, 1978); *I*_nor_ = Shannon’s Diversity Index normalized by sample size, i.e., *I*_nor_ = *I*/ln (sample size).

^b^ Values are given for the entire data set (including 10 loci) and, in parentheses, for the reduced data set (9 loci, excluding Na12_E01b due to a high amount of missing data).

^c^ means calculated only from those populations that have at least four individuals are given in square brackets.

In the Table italic font is just used as a typographic way to distinguish values in the row showing the means from those showing other values.

In the AMOVA, 95% of the total genetic variation was found within populations, and no significant variation between population groups was observed (Table 4). Pairwise F_ST_ values were generally low and ranged from 0.0025 (railway stations *versus* oil mills) to 0.0396 (switchyards *versus* road sections), but all except the lowest ones (railway stations *versus* oil mills) were significant (Table 5).

**TABLE 4:** Three-level AMOVA with six population groups.

Source of variation	degrees of freedom	Sum of squares	Variance components	Percentage of variation^a^
Among population groups	5	94.470	0.00199	0.10^n.s^
Among populations within population groups	29	297.627	0.09212	4.86***
Within populations	3645	6565.500	1.80123	95.03***
*Total*	*3679*	*6957.596*	*1.89534*	

^a^ Significance levels indicated by asterisks: ***p< 0.001, ^n.s^. *p* > 0.05.

In the Table italic font is just used as a typographic way to distinguish values in the row showing the means from those showing other values.

**TABLE 5:** Pairwise F_ST_ values (below diagonal) and their statistical significance (above diagonal).

Population group	RS	SY	RS-B	PO	RO	PF	Varieties
**RS**		0.0078	<0.001	<0.001	<0.001	0.0996	<0.001
**SY**	0.0048		0.0010	<0.001	<0.001	<0.001	<0.001
**RS-G**	0.0046	0.0063		<0.001	<0.001	0.0010	<0.001
**PO**	0.0108	0.0231	0.0140		<0.001	<0.001	<0.001
**RO**	0.0199	0.0396	0.0249	0.0085		<0.001	<0.001
**PF**	0.0025	0.0076	0.0060	0.0113	0.0238		<0.001
**Varieties**	0.0154	0.0285	0.0302	0.0227	0.0265	0.0185	

**FIGURE 3:**
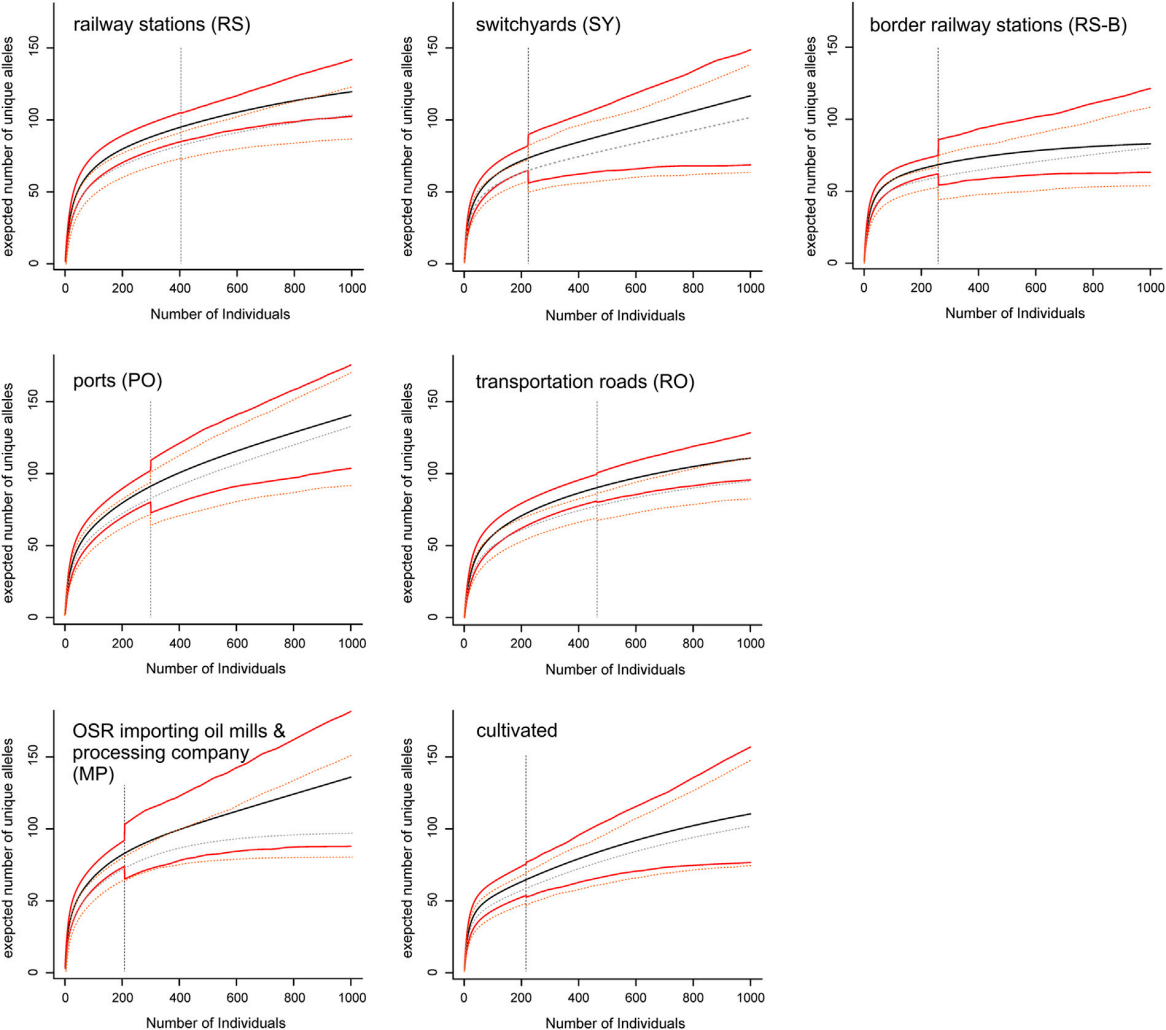
Allelic richness accumulation curves of seven population groups. Mean values (black and grey lines, respectively) of allelic richness and their confidence intervals (red and orange lines, respectively) with increasing sample size are shown for the complete data set (including all ten microsatellite loci; solid lines), and the reduced data set (including all loci but one with a high proportion of missing data; dashed lines). The vertical dashed lines indicate actual sample sizes (i.e., values right to these lines are extrapolated).

Allelic richness (expressed as expected number of unique alleles) of population groups was lowest at border railway stations and highest in ports and in oil mills (Figure 3). Although allelic richness accumulation curves generally flattened out, only in border railway stations the curve became (nearly) saturated, whereas in other population groups (especially switchyards, main Danube ports, oil mills and commercial varieties) no saturation was reached.

**FIGURE 4:**
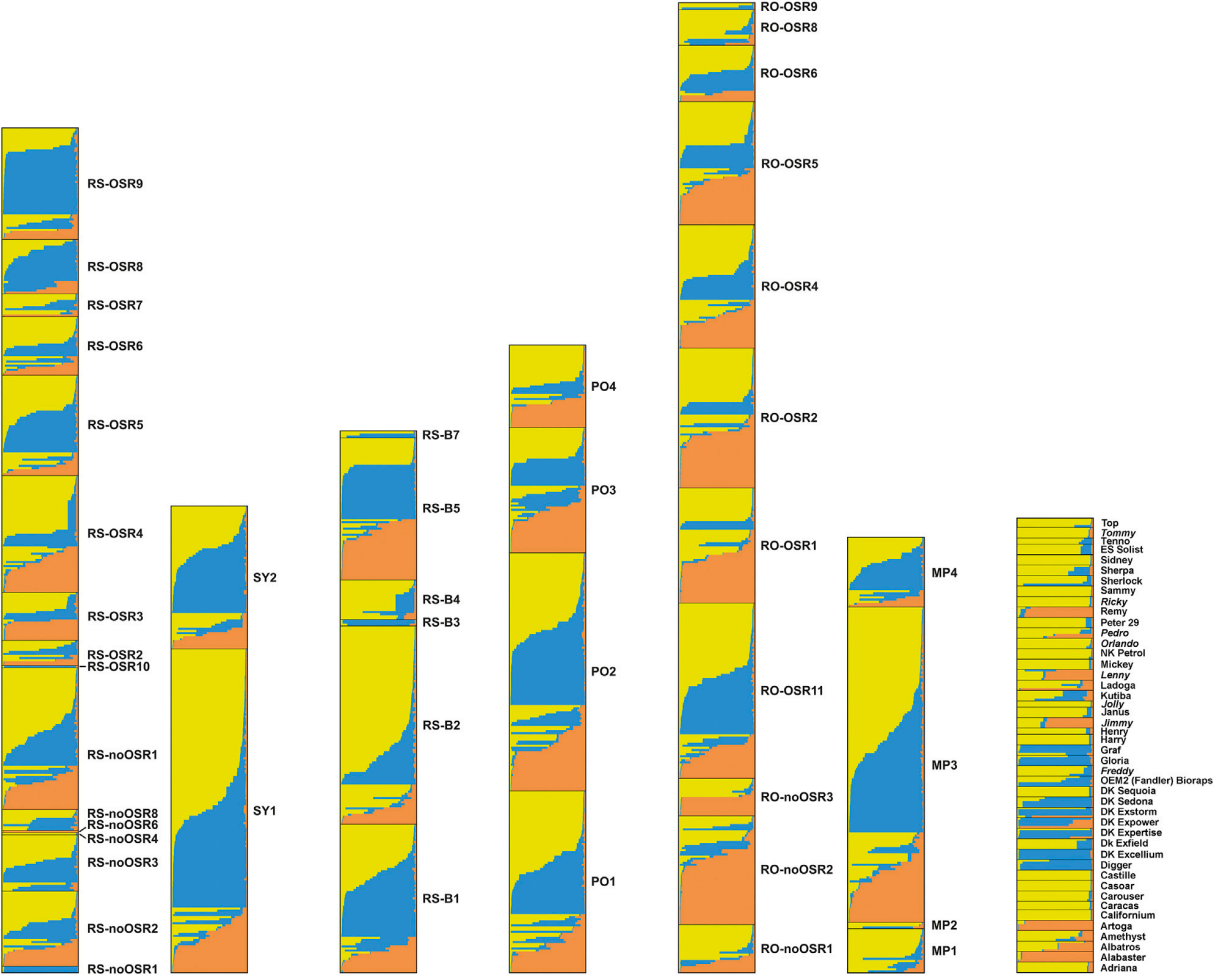
Results of the cluster analysis using Structure for seven population groups. From left to right: railway stations (RS, comprising RS-OSR and RS-noOSR), switchyards (SY), border railway stations (RS-B), ports (PO), transportation roads (RO, comprising RO-OSR and RO-noOSR), OSR importing oil mills and processing company (PF), commercial varieties (non-Austrian commercial varieties from the EC are indicated in italics). Different colours correspond to different genetic clusters. Each horizontal column represents an individual, where the height of the column segments indicates the probability of assignment to the respective genetic cluster.

Applying the DeltaK statistics (Evanno et al., 2005), *K* = 3 was identified as the preferred solution (data not shown). Each of the three gene pools was present in all feral populations (Figure 4). Exceptions were mostly restricted to cases where only few individuals had been sampled (e.g., RS-noOSR1) although a few of the larger populations (with 10 or more individuals) contained essentially only two gene pools (e.g., RS-noOSR8 or RO-noOSR3). All three clusters were present in all tested commercial varieties. Within populations, both individuals with no or nearly no admixture were found alongside individuals with genotypes admixed to different degrees, exceptions being small feral populations as well as commercial varieties, which usually were genetically relatively uniform (i.e., individuals of the same variety had similar, admixed or not-admixed, genotypic composition) (report of Pascher et al., 2016).

## 4 Discussion

The highest levels of genetic variation, especially when considering allelic richness (Figure 3), were observed in the main Danube ports and in oil mills. This result, coupled with the high number of private alleles (more than 50%) at a frequency of about 18% in feral populations (compared to only a single private allele with a frequency of <0.5% in varieties) are consistent with previous assumptions concerning the role of seed spillage along transportation routes as source for establishment of feral OSR populations (e.g., Pivard et al., 2008; Bailleul et al., 2016; Knispel and McLachlan, 2010; Schoenenberger and D’Andrea, 2012). The higher diversity at ports and oil mills compared to switchyards or roads may be the result of regular seed spillage during transport and handling activities and the lack of chemical weed management, so that variable feral populations are more likely to persist.

Overall levels of heterozygosity were roughly similar to those observed in a previous study of commercial varieties and feral OSR populations in Austria based on plant samples collected in 1999–2000 (Pascher et al., 2010). In contrast, genetic diversity in both varieties (mean *I*_*nor*_ 0.137, compared to 0.65) and feral populations (mean *I*_*nor*_ 0.245, compared to 0.76) was markedly lower and thus more homogeneous than previously reported (Pascher et al., 2010). This discrepancy may be due, at least partly, to changes in breeding of the varieties, which are now dominated by hybrid breeds (i.e., lower diversity and lack of private alleles due to the use of the more homogenous F1 parents).

In contrast to earlier studies using similar sets of SSR markers (Elling et al., 2009; Pascher et al., 2010), the between-population component of genetic variation in feral OSR was very low. Two factors may have contributed to this result. Firstly, our sampling focussed on sites where a constant input of kernels during transport is likely, whereas the former studies included less disturbed habitats such as field margins where established feral populations may persist and diverge owing to random genetic drift and selection. Secondly, the feral populations showed similar patterns of genetic differentiation as the commercial varieties; this was evident from the presence of the same three gene pools in admixed and non-admixed individuals (Figure 4). This low number of resolved gene pools contrasts with results of our previous study (Pascher et al., 2010), where we found ten clusters in 18 reference varieties, and further clusters were only represented in feral populations. The reasons for this discrepancy remain elusive, but may include differences in sampling design (established feral populations *versus* newly introduced seedlings); a putatively high genetic heterogeneity of imported OSR, which is obtained in bulk mixtures complying with quality rather than varietal homogeneity; shifts towards more genetically uniform OSR varieties (most varieties proved to be rather genetically uniform, i.e., individuals of the same variety had similar admixed or non-admixed genotypic composition); better correspondence of sampled varieties with the set of actually cultivated varieties, which are expected to be the main source of feral OSR; or methodological issues (low marker-to-sample ratio, conservative nature of the method used by Evanno et al. (2005) to identify the number of *K*).

What can we learn from the results of the present case study on conventional OSR regarding the challenges and performance of handling and tracing genome-edited organisms and, in particular, their unintended contamination of imported conventional kernels? Based on the results of the current study, it is not possible to provide concrete estimates regarding the likelihood, extent, or time scales of seed spillage and spread of accidentally introduced GM OSR seeds into the receiving environment. Rather, the study results allow the identification, description, and evaluation of possible pathways for adventitious introgression of GM OSR seeds, including genome-edited varieties, into Austria, a country, where no GMO has been released so far and import bans on GM OSR varieties are currently in force. Based on these baseline data, preliminary recommendations for the establishment and optimization of post-market environmental monitoring (PMEM) can be derived. In fact, a satisfactory and comprehensive PMEM for introduced seeds of genome-edited OSR varieties has not yet been developed and implemented due to lack of sufficient knowledge and financial resources. In this context, applicable detection methods and their capacity to identify genome-edited modifications are only now in the process of being developed and verified (Fraiture et al., 2022). Another hurdle is the fact that, according to the current European approval regulations, detailed information on GM crop varieties has to be provided by third countries only if the agribusinesses intend to export them into the European Union. The lack of information regarding accidentally introduced genome-edited seeds poses a major challenge to monitoring laboratories in terms of the detection of unknown modifications, which, moreover, may only be single point mutations. Hence, only minimal information about the genome-edited modification and its detection approach is available for Cibus OSR event 5715 despite the data provided in the European GMO database Euginius (www.euginius.eu/: accessed on 23 March 2023). In addition, despite existing European regulations, environmental monitoring has not yet been put into practice in many European countries. The requirements reach so far that operators would be obliged to suppress the regrowth of GM plants that are not approved for cultivation in Europe. However, in order to comply with these requirements, events of genome-edited varieties must first be proven by applying appropriate detection methods. These deficiencies thus affect both the natural environment as a conservation target and the food production chain in terms of consumer choice. In order to guarantee free consumer preference, the products must comply with the current threshold for contamination and must also be labelled. However, this only applies to GM crops that are approved in the European Union. Products containing contaminations with non-approved varieties must be withdrawn from the market in accordance with current EU regulations. Another critical aspect is the issue of coexistence—the unaffected existence of different farming systems (organic, conventional, GMO) in close proximity to each other. In this respect, genome-edited plant seedlings could undermine coexistence requirements if they emerge as volunteers in agricultural fields or as feral plants in natural habitats. Hybridisation with related species could further challenge coexistence requirements.

In our study using OSR as a model system, we were able to show that especially facilities of ports importing kernels from third countries are key entrance points for contamination and represent a source for allowing the formation of novel genotypes. This aspect is of particular importance in the case of accidental contamination of non-GM kernels with genome-edited seeds imported from countries such as the US, Canada, and Australia, where genome-edited seeds are considered non-GMO and are therefore not labelled. It is entirely unknown which specific OSR varieties are imported into or transported through Austria which is a crossroads for flow of wares located in the middle of Europe (Pascher et al., 2017). According to personal communications with managers of port facilities, railroad stations, and warehouses (Pascher et al., 2016), all OSR varieties are transported from exporting countries to Austria as mixtures of different varieties. These varieties must meet certain quality standards such as certain oil content, low content of erucic acid and glucosinolates, and are not bred using genetic engineering techniques. Consequently, the identities of origin and traceability of certain varieties are not given. For confidentiality reasons, it was not possible to receive variety-specific data from Statistics Austria (http://www.statistik.at/; accessed on 23 March 2023) nor from the Austrian Federal Ministries (IACS/INVEKOS, Austria data: https://info.bml.gv.at/themen/landwirtschaft/eu-agrarpolitik-foerderungen/direktzahlungen/Invekos.html; accessed on 24 March 2023). At the time of sampling, Hungary, Serbia, and Slovakia were the primary exporters of OSR to Austria. In contrast to reliable data for OSR transport via ship, comparatively imprecise data were available concerning transport via train or truck. For example, routes are not specified for truck drivers to transport kernels to oil mills. This impedes the traceability and detection of any seed loss. Consequently, major information gaps exist regarding the identity of the origin and the traceability of accidentally introduced genome-edited seeds. Based on the results of our model study, we identify sensitive stages and sites in the transport and processing chain regarding seed losses of imported OSR. We find that kernel import, loading and processing facilities for OSR are hotspots for seed spillage and require special attention in case of contamination of conventional kernels with genome-edited seeds in the context of PMEM. According to the results of our study, the hotspots for seed loss are mainly port facilities and oil mills.

## 5 Conclusion

As stipulated in the European regulations, a comprehensive environmental risk assessment is required also for genome-edited seeds. In addition, the PMEM of OSR imports must be designed and set up in such a way that contamination with unauthorised genome-edited seeds can be detected in deliveries of imported kernels. We, therefore, provide the following recommendations: When developing and implementing such a PMEM, commodity flows should be identified. As a first step and depending on the available financial resources, local inspection and testing of feral plants should be focussed on kernel handling and processing sites including ports of entry or transhipment ports and oil mills. The provision of reference material as well as the availability and preparedness of appropriate methodological detection methods are, however, the basic prerequisites for identifying point mutations caused by artificial intervention. Microsatellites, such as those used in the present study, may be applied to identify polymorphism to hold responsible agribusinesses accountable for violations in the case of liability concerns. Using accurate detection methods, the detection, traceability, and labelling of genome-edited organisms could then continue to be enforced in the same way as for classical GMOs, which would allow for regulation in the European Union under Directive 2001/18/EC. The regulations should continue to be followed, in compliance with the precautionary principle, a robust ecological risk assessment and post-market environmental monitoring.

## Data Availability

The original contributions presented in the study are included in the article/Supplementary Material, further inquiries can be directed to the corresponding author.
