# Supplementary material for: Contamination of imported kernels by unapproved genome-edited varieties poses a major challenge for monitoring and traceability during transport and handling on a global scale: inferences from a study on feral oilseed rape in Austria
Source: Front Genome Ed. 2023 Apr 20;5:1176290. doi: 10.3389/fgeed.2023.1176290 (PMC10156978; doi:10.3389/fgeed.2023.1176290)
Supplement: Supplementary file 1 [file DataSheet1.pdf]

Supplementary material

S1 Table. Relevant hybridisation partners of oilseed rape registered at the 60 sample sites.

Species	Number of registered sample sites
Brassica napus (feral)	1
Brassica rapa (feral)	5
Diplotaxis tenuifolia	20
Erucastrum gallicum	4
Raphanus raphanistrum	7
Sinapis alba	17
Sinapis arvensis	21
Sisymbrium loeselii	15
Sisymbrium officinale	17
Sisymbrium orientale	3
Sisymbrium strictissimum	1

S2 Table. Locus by locus descriptive statistics for commercial varieties and feral populations.

Locus Name	Allele Length	Sample Size	n_a	n_e	H_O	H_E	I_{nor}	AR	No. Priv. Alleles	No. Alleles
**Commercial varieties**										
Na12_A08	151–183	217	9	1.846	0.194	0.459	0.183	7.406 (8.916)	0	9
Na12_C06	206–212	217	4	2.084	0.180	0.521	0.169	3.788 (3.999)	0	4
Na12_E01a	222–236	217	7	3.132	0.184	0.682	0.225	6.320 (6.998)	0	7
Na12_E01b	246–258	117	5	1.862	0.034	0.465	0.192	5.000	0	5
Na12_C08	278–346	217	13	4.172	0.341	0.762	0.315	11.537 (12.943)	0	13
NA12_Eo6a	103–125	215	7	1.365	0.223	0.268	0.111	5.862 (6.962)	0	7
Na12_C12	240–256	212	7	1.633	0.127	0.389	0.162	6.544 (6.995)	0	7
Na10_C01a	213–215	217	2	1.042	0.041	0.041	0.019	1.999 (2.000)	0	2
Na10_C01b	247–259	217	4	1.113	0.060	0.102	0.046	3.537 (3.972)	0	4
Na12_D11	342–356	211	7	1.311	0.104	0.238	0.103	6.157 (7.000)	1	7
Sum									*1 (1)*	*65 (60)*
Mean		*205.7 (215.6)*	*6.5 (6.7)*	*1.956 (1.966)*	*0.149 (0.162)*	*0.393 (0.385)*	*0.152 (0.148)*	*5.815 (6.643)*	*0.1 (0.11)*	*6.5 (6.67)*
S.D.		*31.2 (2.4)*	*3.1 (3.2)*	*0.986 (1.045)*	*0.095 (0.092)*	*0.234 (0.247)*	*0.087 (0.095)*	*2.597 (3.182)*	*0.32 (0.33)*	*3.06 (3.2)*

**Feral populations**										
Na12_A08	147–187	1853	16	2.204	0.238	0.546	0.163	9.184 (10.404)	7	16
Na12_C06	192–234	1853	18	2.987	0.185	0.665	0.162	7.075 (8.856)	14	18
Na12_E01a	220–242	1854	12	3.279	0.217	0.695	0.176	6.424 (7.208)	5	12
Na12_E01b	244–274	888	11	2.693	0.064	0.629	0.193	8.967	6	11
Na12_C08	264–348	1845	29	6.004	0.409	0.834	0.279	14.525 (16.116)	16	29
NA12_Eo6a	103–129	1833	11	1.657	0.216	0.397	0.108	6.475 (7.671)	4	11
Na12_C12	228–280	1850	16	1.749	0.100	0.428	0.129	9.255 (10.695)	9	16
Na10_C01a	213–217	1855	3	1.044	0.018	0.042	0.014	2.058 (2.114)	1	3
Na10_C01b	231–277	1852	9	1.063	0.016	0.060	0.023	3.784 (4.648)	5	9
Na12_D11	338–354	1792	9	1.198	0.060	0.165	0.059	6.718 (7.763)	3	9
Sum									*70 (64)*	*134 (123)*
Mean		*1747.5 (1843.0)*	*13.4 (13.7)*	*2.388 (2.354)*	*0.152 (0.162)*	*0.446 (0.426)*	*0.131 (0.124)*	*7.447 (8.386)*	*7 (7.11)*	*13.4 (13.67)*
S.D.		*302.6 (20.3)*	*7.0 (7.3)*	*1.501 (1.588)*	*0.124 (0.127)*	*0.278 (0.287)*	*0.082 (0.086)*	*3.396 (3.952)*	*4.76 (5.04)*	*6.98 (7.35)*

n_a = observed number of alleles; n_e = effective number of alleles [Brown and Weir 1983]; H_O = observed heterozygosity; H_E = unbiased expected heterozygosity [Nei 1978]; I_{nor} = Shannon's Diversity Index normalized by sample size, i.e., $I_{nor}=I/\ln(\text{sample size})$; AR = Allelic Richness calculated using the rarefaction method of [El Mousadik and Petit 1996]; No. Priv. Alleles = number of private alleles; No. Alleles = total number of alleles. Values are given for the entire data set (including 10 loci) and, in parentheses, for the reduced data set (9 loci, excluding Na12_E01b due to a high amount of missing data).

References

Brown, A. H., and Weir, B. S. (1983). “Measuring genetic variability in plant populations” in *Isozymes in plant genetics and breeding Part A*, Editors S. D. Tanksley and T. J. Orton (Elsevier), 219–239.

El Mousadik, A., and Petit, R. J. (1996). High level of genetic differentiation for allelic richness among populations of the argan tree (*Argania spinosa* (L.) Skeels) endemic to Morocco. *Theor. Appl. Genet.* 92, 832–839. doi:10.1007/BF00221895

Nei, M. (1978). Estimation of average heterozygosity and genetic distance from a small number of individuals. *Genetics* 89, 583–590. doi:10.1093/genetics/89.3.583
